# The complotype: dictating risk for inflammation and infection

**DOI:** 10.1016/j.it.2012.06.001

**Published:** 2012-10

**Authors:** Claire L. Harris, Meike Heurich, Santiago Rodriguez de Cordoba, B. Paul Morgan

**Affiliations:** 1 Institute of Infection and Immunity, School of Medicine, Cardiff University, Heath Park, Cardiff, CF14 4XN, UK; 2 Centro de Investigaciones Biológicas (CSIC) and Ciber de Enfermedades Raras, Ramiro de Maeztu 9, 28040 Madrid, Spain

**Keywords:** complement, complotype, inflammation, alternative pathway

## Abstract

Complement is a key component of immune defence against infection; it potently drives inflammation at sites of pathology and is essential for killing of pathogens. Genetic linkage of common complement polymorphisms to disease has advanced the concept that subtle changes in complement activity significantly affect disease risk. Functional analyses of disease-linked polymorphic variants demonstrate that, although individual polymorphisms cause only small changes in activity, when combined, the aggregate effects are large. The inherited set of common variants, the complotype, thus has a major impact on susceptibility to inflammatory and infectious diseases. Assessing the complotype of an individual will aid prediction of disease risk and inform intervention to reduce or eliminate risk.

## Complement system

Complement is a major component of innate immunity and offers a first line of defence against infection by triggering inflammatory responses to alert the immune system to impending danger. Complement plays crucial roles in targeting and killing microbes and infected cells, flagging them for phagocytic clearance and punching holes through cell membranes to cause lysis. Its role extends beyond fighting infection; it is also required for efficient clearance of apoptotic cells and immune complexes as well as, perhaps more surprisingly, modulating adaptive immune responses. Complement is a proteolytic cascade, triggered via three activation routes, the classical pathway (CP), the lectin pathway (LP) and alternative pathway (AP); together, these recognise almost any potential target (reviewed in [1]).

During the past decade, several studies have associated genetic variations in complement components and regulators with various chronic and infectious diseases. The recent functional characterisation of these complement protein variants has provided important insights into the pathogenic mechanisms involved in complement-related disorders. Here, we review evidence linking complement polymorphisms with disease, and discuss recent research that identifies the functional mechanisms behind genetic linkages in the complement AP. We introduce the concept of the ‘complotype’, which represents the pattern of genetic variants in complement genes inherited by an individual, which alters risk for both inflammatory disorders and infectious diseases involving complement. Recent evidence suggests that the complotype also extends beyond the AP to include both activating and control proteins from other complement pathways. A detailed understanding of the complotype will aid prediction of disease susceptibility and assessment of current disease status, and inform decisions on therapeutic interventions.

## Key role of AP convertase

The key steps during complement activation involve formation of labile enzymatic complexes named C3-convertases [C3bBb (AP); C4b2a (CP and LP)], which cleave C3 to generate C3b. The AP C3 convertase is crucial for efficient complement activation, regardless of the initial trigger, because it amplifies the response and is intrinsically active, in contrast to the CP and LP C3-convertases for which activation is triggered by immune complexes and bacterial mannose groups respectively (reviewed in [2]) (Box 1). Continuous production of activated C3, either C3(H_2_O) produced by spontaneous hydrolysis of the internal thioester, or C3b generated by convertases or other plasma proteases in plasma, is known as ‘tickover’ [3]. This ensures low-level generation of AP convertase, necessary for depositing C3b onto surfaces (Box 1). Thus, the AP is ‘primed’ for immediate activation and amplification, leading to C3b deposition on the target pathogen and ‘foreign’ surface. Tickover activation is stringently controlled by factor H (fH), which, in collaboration with the plasma serine protease factor I (fI), inactivates C3b and C3 convertases [4,5]. fH is an abundant plasma protein (0.2–0.5 g/l) with an elongated structure presenting several binding sites for C3b and glycosaminoglycans (GAGs) along its length. The GAG-binding domains are key for protection of self tissues because they locate fH to cell membranes where it dissociates deposited AP convertases and, together with fI, inactivates C3b. FH is a member of a gene family that includes five homologous fH-related proteins (FHR1–5) (reviewed in [5]). These may also have AP regulatory roles analogous to fH; alternatively, they may act as competitors to inhibit fH regulation [6]. Self tissues are also protected by membrane-bound proteins that restrict complement activation by either acting as cofactor for proteolytic inactivation of C3b by fI or by accelerating convertase dissociation (Box 1) (reviewed in [7,8]). Thus, in health, spontaneous activation of C3 in plasma is kept at a low level and further complement activation and C3b deposition is restricted to targets lacking surface regulators.

## Complement AP dysregulation and inflammatory disease

In health, complement is in homeostatic balance. Tickover confers the capacity for rapid activation and amplification on pathogens, which is essential for efficient clearance but is risky in the sense that any disturbance in homeostasis can provoke damage to self. Dysregulation of the central components of the AP amplification loop, C3, factor B (fB), factor D (fD), or the control proteins, fH and fI, might therefore be predicted to affect plasma AP activity, thereby affecting risk of acute or chronic inflammation and disease.

Common polymorphisms in AP protein genes, including those encoding C3, fB and fH, are associated with disease risk (Box 2), although the underlying mechanisms have been unknown. The best example of a disease strongly linked to AP polymorphisms is age-related macular degeneration (AMD). End-stage (blinding) AMD affects 2% of those aged over 50 years, with the incidence rising steeply with age and approaching 20% in those over 85 years. Annual cost to the UK economy exceeds £5 billion [9]. Retinal drusen, the hallmark of AMD, is known to contain complement proteins, but little was made of this until 2005 when several papers linked the common H1 haplotype of the fH gene (*CFH*), including the fH_Y402H_ polymorphism (rs1061170), with AMD. Two copies of the risk haplotype increased odds of developing AMD seven fold [10–12]. A second *CFH* haplotype, H2, was protective for AMD; this haplotype includes the fH_V62I_ variant [rs800292; odds ratio (OR) 0.54] [13]. The H4 haplotype that carries a deletion of the genes encoding the FHR proteins 1 and 3 (*CFHR1*, *CFHR3*), is protective for AMD (OR 0.48) [14] and this deletion is common in Caucasians (null allele frequency 20%). A common allelic variation in the *CFHR1* gene is the FHR1*A variant that differs from FHR1*B by three amino acids [15]. CFHR1*A is risk for AMD (OR 2.08) and in strong linkage disequilibrium (LD) with the *CFH-402H* allele [16]. AMD associations are also reported for variants in genes encoding key AP proteins that either promote or control the AP amplification loop (Box 1). These include fB (fB_R32Q_, rs641153; protective, OR 0.32) [17], and C3 (C3_R102G_, rs2230199; risk, OR:2.6) [18]; a recent targeted genotyping approach identified a single nucleotide polymorphism (SNP) just 3′ of the gene encoding fI (*CFI*) that is associated with reduced AMD risk [19].

Dense deposit disease (DDD) is a rare cause of renal failure associated with copious complement deposition in the kidney and systemic activation of AP components (reviewed in [20]). Clinically, DDD is linked to AMD – most DDD patients develop eye changes typical of AMD at a comparatively young age. The *CFH* haplotypes associated with increased (H1 including rs1061170; fH_Y402H_) and decreased (H2, including rs800292; fH_V62I_) risk of AMD conferred similar effects on DDD risk [21]. C3 polymorphisms (C3_R102G_ and C3_P314L_) also increase risk of DDD and, importantly, DDD risk increases dramatically when two or more risk alleles in fH and C3 are present in an individual [22].

Atypical haemolytic uremic syndrome (aHUS) is a rare, life-threatening, disease characterised by a triad of thrombocytopenia, microangiopathic haemolytic anaemia and acute renal failure with abundant renal complement deposits [20]. Of the AP polymorphisms linked to AMD and DDD, only fH_V62I_ influences risk of aHUS [21]. The fH_Y402H_ and C3_R102G_, variants, which are risk factors in AMD/DDD, do not affect aHUS, and the *CFHR1/CFHR3* deletion that is protective in AMD, is risk for aHUS, because lack of FHR1 is strongly associated with serum anti-fH autoantibodies [15]. Two other common haplotypes in *CFH* and *MCP* are strong aHUS risk factors [23,24].

Over half of aHUS patients have point mutations in AP proteins, commonly in fH, these are clustered in the carboxy terminus and disrupt the capacity of fH to bind to surfaces [25,26]. Other complement protein mutations also associate with aHUS risk, including gain-of-function mutations in fB and C3 [27,28]. These mutations cause AP dysregulation by either enhancing convertase formation/stability or disrupting convertase regulation (reviewed in [29]); for each, penetrance is incomplete and co-inheritance of risk-conferring polymorphisms in the same or different AP proteins markedly increases odds of developing disease [24,30].

The fact that many of the variants affecting susceptibility to AMD/DDD have no effect on risk of aHUS and vice versa implies that these diseases of complement dysregulation have different underlying mechanisms [26,29]. The current understanding is that aHUS occurs when the capacity to prevent inappropriate AP activation on cell surfaces is impaired, whereas AMD/DDD occurs when AP fluid-phase regulation is impaired. Several groups have used plasma complement proteins as biomarkers in AMD for early detection, disease monitoring or to guide intervention; elevated plasma levels of activation fragments C3a, C3dg, C5a, and Bb, terminal complement complex (TCC), and fD are potential AMD biomarkers and can similarly be used to anticipate aHUS recurrences [31–33].

## Identifying functional consequences of complement AP polymorphisms

**Figure 1:**
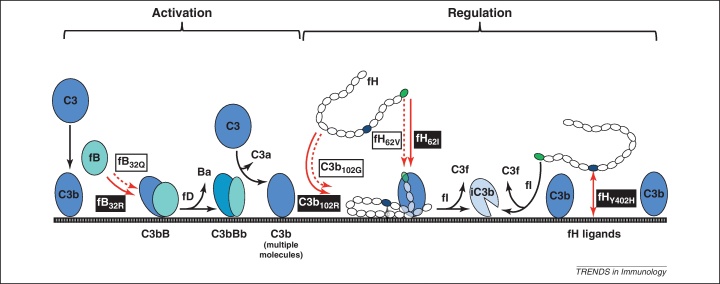
Functional effects of complement protein polymorphisms. C3b generated by tickover routes binds surfaces through its thioester. C3b captures factor B (fB) to form the alternative pathway (AP) pro-convertase (C3bB). Factor D (fD) cleaves fB in the pro-convertase to yield C3 convertase (C3bBb); this cleaves more C3 to C3b that binds membranes, captures fB and builds more convertase (the AP amplification loop). The plasma AP regulator factor H (fH) comprises 20 short consensus repeats (SCRs); fH binds C3b in the convertase, displacing Bb to inactivate the convertase. fH bound to C3b is also a cofactor for cleavage of C3b by factor I (fI) to yield the inactive product iC3b. Complement polymorphisms affect AP activation/regulation in different ways (red arrows). The fB_R32Q_ modulates fB affinity for C3b in convertase formation; fB_R32_ binds better so makes more pro-convertase, more convertase and more AP activation (stronger binding illustrated by solid red arrow). C3_R102G_ affects affinity of C3b for fH; C3b_102G_ binds fH less strongly (weaker binding illustrated by broken red arrow), leading to less efficient C3b inactivation by fI, and thus drives more AP activation. fH_V62I_ (in SCR1, green) also affects C3b-fH binding; fH_62I_ binds C3b more strongly (unbroken arrow) so better regulates the convertase, facilitates C3b cleavage and inhibits AP activation. These three AP polymorphisms, influencing AP activity in different ways, collaborate to set AP activating capacity. Other disease-associated AP polymorphisms also affect activity; for example, the AMD-associated fH_Y402H_ polymorphism (in SCR7, blue) probably alters capacity of fH to bind surfaces in the eye, influencing local convertase regulation.

Despite dramatic effects on disease susceptibility, the underlying mechanisms by which common polymorphisms in AP proteins alter disease risk have remained unclear. The situation is further complicated by the fact that the *CFH*, *CFHR3* and *CFHR1* genes comprise a major susceptibility locus including one haplotype conferring increased risk (H1) and two that protect (H2, H4) from AMD. Although fH_402H_, fH_62V_ and Δ*CFHR3–CFHR1* have been implicated as the responsible genetic variants within respective haplotypes, any of the polymorphisms in that haplotype could be functionally responsible, making it difficult to pinpoint the culprit. To address this issue, a biochemical approach has been used to identify how polymorphisms predispose to disease. AP protein variants were purified to homogeneity from healthy, homozygous donors and the effects of these single amino acid changes on AP activity tested. The AMD-linked polymorphism in fB (fB_R32Q_) is in the Ba domain that is released during formation of the active enzyme C3bBb. Nevertheless, the risk variant, fB_32R_, was more lytic in complement functional assays due to higher affinity for C3b compared to fB_32Q_, which gives rise to enhanced formation of proenzyme, C3bB, and increased formation of active convertase (Figure 1a) [34]. The fH_V62I_ polymorphism altered the capacity of fH to regulate C3b; fH_62I_ bound better to C3b, resulting in enhanced cofactor activity and increased formation of iC3b (Figure 1b) [35,36]. Association of the common C3 polymorphism (C3S/F; C3_R102G_) with many diseases, including AMD, has been recognised for decades but the molecular basis of disease association has been enigmatic. The purified AMD risk variant, C3_102G_, bound fH less strongly than C3_102R_ and was less efficiently inactivated, thereby increasing AP activity (Figure 1c) [37]. All three polymorphisms directly influence the AP convertase, although each alone had a relatively small effect, for example, the K_D_ of the fH_62V_ variant for C3b binding was 1.3 μM, and that of the fH_62I_ variant was 1.0 μM. When combined, the functional effect of the variants was greatly enhanced; this is because all three proteins promote or control the amplification loop, which is key for amplifying small triggers to give massive downstream effects (Box 1). In assays that simultaneously interrogated the effects of polymorphisms in all three proteins on convertase activity, AMD risk variants together were sixfold more active than the combined AMD protective variants [37]. Even in an AP haemolytic assay (AH50), a clinical test which does not fully interrogate fH function, the combination of three AMD-risk variants yielded whole serum with twice the complement haemolytic activity of serum containing protective variants. This dramatic variability in AP activity will inevitably influence tissue damage and inflammation wherever complement is activated. In support of these data, combined inheritance of DDD risk polymorphisms in fH and C3 was associated with higher AP activity in genotyped healthy controls [22].

The mechanism by which the fH_Y402H_ polymorphism affects disease risk is unresolved, but there is no evidence to suggest that it is due to altered AP convertase regulation (Figure 1d). Differential binding to C-reactive protein (CRP), an acute phase reactant that binds to damaged cells has been described [38,39]. Other studies suggest differential binding to heparin and other polyanionic ligands [40–42]; indeed, *ex vivo* binding studies in AMD retina have shown preferential binding of fH_402Y_ to Bruch's membrane that is dependent on sulfated GAGs [43]. Recently, malondialdehyde (MDA), a lipid peroxidation product abundant in AMD retina, has been shown to bind fH with a strong selective preference for fH_402Y_, provoking the suggestion that failure to recruit fH_402H_ to sites of photo-oxidation damage causes local complement dysregulation and pathology in AMD [44]. The recent demonstration that the CFHR1*A allotype, which is risk for AMD, is in strong LD with *CFH402H* adds an additional possibility – that the strong association of AMD risk with the H1 haplotype is functionally unrelated to the *CFH402* polymorphism [16].

## The complotype

Together, the inherited repertoire of common polymorphisms in genes encoding complement proteins and regulators affects the delicate balance between activation and regulation and sets an individual's intrinsic complement activity; this repertoire can be referred to as the complotype. This term was originally coined to describe haplotypic combinations of genetic variants of the MHC-linked complement genes [45], but here it is used in the broader context of the whole complement system. The data discussed so far show that inheritance of more active variants of AP components (C3, fB) or less active variants of regulators (fH, fI, MCP) swings the balance in favour of AP activation and inflammation, whereas inheritance of less active components and more active regulators dictates less AP activation and inflammation. Table 1 illustrates theoretical values for different combinations of alleles in the Caucasian population based on individual allele frequencies. We predict that those combinations leading to a more active complement system put an individual at risk for inflammation, whereas those combinations at the other end of the scale are risk for infection.

An additional dimension is provided by inherited variability in the expression levels of AP proteins. Thus, functional and expression level differences in AP proteins collude to set AP activating capacity, influencing susceptibility to diseases involving complement. Each of these disease-associated variants is common in the population and, individually, their effect is small. Indeed, genetic studies in AMD and aHUS show that multiple hits, polymorphisms and mutations, in AP components are needed to cause pathology. Penetrance of aHUS in carriers of a disease-associated AP protein mutation is approximately 50%, but increases considerably if the individual also carries one or more additional disease-associated mutation or risk polymorphisms altering function or expression of another AP protein [24,27]. Whether a variant is risk or protective depends on the underlying disease trigger and mechanisms leading to pathology.

So far, this review has focused on three prototypical diseases of complement dysregulation, AMD, DDD and aHUS; however, many other diseases are also influenced by these same polymorphic variants; for example, fH_402H_ is risk for myocardial infarction and cardiovascular disease [46], fB_32Q_ is risk for some infections and disproportionately represented in multiple sclerosis and type I diabetes [47–49], whereas C3_102G_ is risk for not only DDD [50], but also renal allograft rejection [51], IgA nephropathy [52] and systemic vasculitis [53].

## The complotype extends beyond the AP

In developing the concept of the complotype, we have focussed on AP components (C3, fB) and regulators (fH, fI, MCP, FHR proteins). However, the complotype encompasses polymorphisms in other complement proteins that are associated with disease and probably affect aspects of complement activity.

Genome-wide association studies (GWAS) in Alzheimer's Disease (AD) have identified polymorphisms in two other genes encoding proteins associated with the complement system, complement receptor 1 (CR1; CD35) and clusterin, that independently influence risk of developing AD [54,55]. CR1 promotes fI cleavage of C3b to form iC3b and C3dg, which are ligands for CR3 and CR2, respectively; the latter being crucial for efficient B cell response to antigen [56]. CR1 also plays a major role in erythrocyte-mediated immune complex clearance [57]. The associated SNPs (rs6656401 and rs3818361) are intronic and effects on CR1 activity or expression are unknown. A recent report describes an AD-linked coding polymorphism in the *CR1* gene (rs4844609; S1610T) suggested to influence binding of C1q and mannan-binding lectin (MBL) [58]. Clusterin is a plasma protein that binds fluid phase C5b–7, thereby preventing formation of membrane attack complex (MAC) on cells [8]. Clusterin also binds dead and dying cells and tissue debris, is abundant in plaques in AD brains, and increased plasma clusterin is a biomarker for AD disease severity and clinical progression [59]. Two intronic SNPs in the *Clu* gene, rs9331888 and rs1136000, show strongest association with AD; the former influences the pattern of alternative splicing in the *Clu* gene [60], and is associated with low plasma clusterin levels in AD patients and controls [61].

Several studies have described association of SNPs (rs10818488, rs2416808) in a region of chromosome 9q that encompasses genes for TNF receptor-associated factor (TRAF)1 and complement C5 with risk for rheumatoid arthritis (RA), [62] and coeliac disease [63]. Both *TRAF-1* and *C5* are excellent candidate genes for perpetuating inflammation in RA; however, other evidence implicates the complement terminal pathway in RA pathogenesis, including identification of TCC in diseased joints, joint fluid and plasma of RA patients [64]. Terminal pathway activation is also implicated in osteoarthritis [65], but no complotype studies have been reported; this age-associated, common chronic inflammatory disease is a prime candidate for future analysis of the role of the complotype in susceptibility or severity. Other SNPs in C5 have been linked to renal allograft survival [66]; one of four haplotype blocks spanning 4 *C5* SNPs is associated with decreased graft function at 1 and 7 years post-transplant. Serum C5 levels are not different between the haplotypes. Intriguingly, a nonsynonymous coding SNP (rs1926447; T325I) in the gene for carboxypeptidase B (CPB), a plasma inhibitor of C5a activity, is associated with decreased risk of severe RA [67]; the ‘protective’ CPB variant displays increased plasma half-life and C5a-inhibiting activity, suggesting a mechanism for the observed protection. Although SNPs have been described in each of the other terminal components, no firm disease associations have yet been reported. Intriguingly, heterozygous deficiency of C9, very common in the Japanese population, has recently been shown to reduce by 4.7-fold the risk of developing neovascular AMD, emphasising the importance of the terminal pathway in this disease [68].

## The complotype and infection

Functional studies and links to chronic inflammation demonstrate that an individual inherits a complotype that places him/her at a point on a spectrum of complement activity from high to low. Individuals at the high end of the complotype spectrum are more prone to chronic inflammation; at the opposite end of the spectrum, individuals with a low activity complotype are protected from chronic inflammation, but at the cost of increased susceptibility to infection. A low activity complotype would thus be a negative selective pressure; in support of this, those variants that constitute the less active complotype are much less prevalent (see theoretical values in Table 1). Indeed, in sub-Saharan African populations at high risk of death from infection there is further skewing of the complotype towards a more active complement system [69]. A striking experiment of nature surrounded the sad fate of 19th century Dutch immigrants to Surinam; soon after arrival the community was devastated by typhoid and yellow fever, wiping out 60% of their number. Descendants of the survivors showed much higher frequency of the most active C3 allele, C3_102G_, compared to the source Dutch population; evidence that selective pressure from infection acutely selected this infection-protective C3 variant [70].

Evidence that the complotype directly influences infection risk has emerged from recent genetic studies. A GWAS for meningococcal disease has identified risk SNPs in *CFH* and *CFHR3* [71]. The coding SNP in *CFH*, (rs1065489; E936D) has been confirmed in a replication study, with the D allele being protective. Interestingly, meningococcal disease incidence differs between ethnic groups and inversely parallels frequency of the protective allele [72]. The associated SNP in *CFHR3* is in LD with rs1065489 and could plausibly be causative. However, a targeted genotyping approach has revealed a polymorphism in a putative nuclear-factor (NF)-κB responsive element in *CFH*, which probably decreases expression of fH, to be associated with meningococcal disease [73]. It is notable that this NF-κB polymorphism, which is protective for meningococcal disease and in the same haplotype as rs1065489 (E936D) [23], is also associated with risk for aHUS [23]. Reduced levels of fH, a disadvantage for cell surface protection and therefore risk for aHUS, may be protective for meningococcal disease as fH is ‘hijacked’ for complement protection by *Neisseria meningitidis* through binding a bacterial surface receptor, fHbp [74]. Whether or not reduced binding of fH is the protective mechanism, these findings strongly implicate complement control in setting disease risk. It is likely that the whole complotype will contribute to risk in meningococcal disease – variation in host-activating proteins will alter the rate or levels of C3b opsonisation, and variation in activity or levels of key host control proteins will not only control opsonisation rate but also affect sensitivity to lysis and protection afforded by hijacked fH. A common coding polymorphism in complement C5, rs17611 (V802I), was recently associated with unfavourable outcome in bacterial meningitis (OR, 2.25; 95% CI, 1.33–3.81; *P*=0.002) in a Dutch prospective study [75]. The effect of this polymorphism on C5 function is unknown.

## Concluding remarks

This review presents evidence that common polymorphisms in complement proteins dictate complement activity in an individual with resultant effects on susceptibility to inflammatory and infectious diseases. Although initially focussed on the AP and a small group of diseases driven by AP dysregulation, the complotype encompasses the entirety of the complement system and the list of diseases affected by complotype grows steadily as genetic studies implicate known or novel complement polymorphisms. Even in these few diseases of AP dysregulation, the different patterns of linked polymorphisms highlight the fact that complement is activated in different ways and at different sites dictated by the complotype to cause different pathologies. In some diseases the importance of the complotype is revealed by its effect on disease penetrance in the presence of a causative mutation.

We predict that analysis of an individual's complotype will be widely used, in conjunction with environmental and other known risk factors, to predict disease risk and course [16,31–33]. Genetic complotype analyses will provide relevant data, but measurement of polymorphic variants at the protein level using specific monoclonal antibodies is rapid and will add data on plasma levels [76]. Measurement of complement activation products, of proven value in AMD, will provide additional information, particularly valuable in acute conditions. Multiplexed complotype assays that measure all relevant complement analytes will enable rapid and efficient complotyping of individuals at risk, and identify those most likely to benefit from interventions – either modifying other risk factors or directly targeting complement using agents selected based on the complotype. These measures will have a major impact on morbidity and mortality in the many diseases involving complement dysregulation.

## Disclaimer statement

S.R.deC. has undertaken consultancy work for Alexion and is listed as a co-inventor on a patent held by Secugen SL, the Agencia Estatal Consejo Superior de Investigaciones Cientificas and the University of Navarra, regarding a method for the prediction of risk of developing AMD in the Spanish population (Spain Patent No.: P201131516). B.P.M. has undertaken consultancy work for Baxter, Viropharma and Alexion. B.P.M. and C.L.H. are named on a patent held by Cardiff University that protects monoclonal antibodies specific for the Y402H variants of factor H. None of these interactions has influenced the results and interpretations in this article.

## Figures and Tables

**Figure I:**
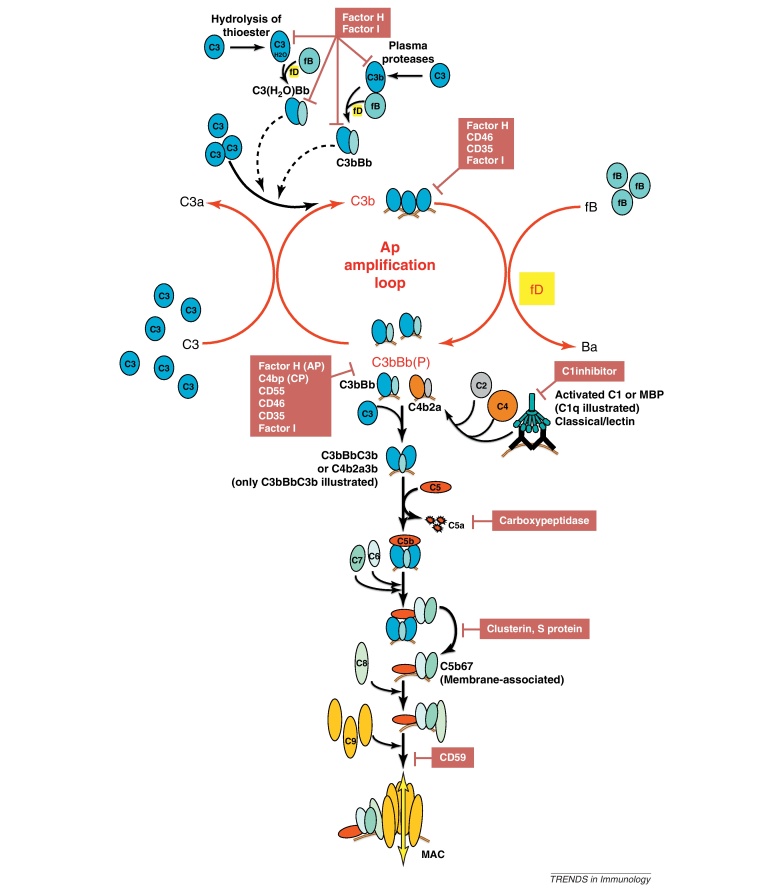
Central role of AP convertase in complement activation. Complement tickover occurs through hydrolysis of the C3 thioester, or cleavage of C3 to C3b by plasma proteases. Fluid phase production of either molecule results in formation of the AP C3 convertase, C3bBb, and production of further C3b which either binds a surface or remains fluid phase. Each newly produced C3b can in turn form a convertase, which cleaves C3, resulting in exponential production of C3b. This self-propagation, referred to as the ‘amplification loop’ and indicated here in red, is responsible for amplifying a small trigger to yield large responses. C3b formed through any activation pathway feeds into the amplification loop. Binding of C3b to C3 convertase creates C5 convertase; cleavage of C5 and generation of C5b marks the start of the terminal pathway. C6 and C7 bind C5b to form C5b67, which is released from convertase, binds membrane and incorporates C8 and multiple C9 molecules to form the MAC. Self tissues are protected from accidental complement damage by regulatory proteins present in plasma and on membranes, indicated by maroon boxes. Note the abundance of regulators which control the amplification loop and C3 convertases.

**Table 1:** Combined allele frequency of common polymorphic variants in C3_R102G_ (rs2230199; single allele frequency R/G: 0.8/0.2), fB_R32Q_ (rs641153; R/Q: 0.765/0.11), and fH_V62I_ (rs800292; V/I: 0.79/0.21) and their expected prevalence in the Spanish population .

Allele Combination	% Frequency	Prevalence in Caucasians
C3102RR, fB32RR, fH62VV	23.4	4 (i.e., 1 in 4)
C3102RR, fB32RR, fH62VI	12.4	8
C3102GR, fB32RR, fH62VV	11.7	9
C3102RR, fB32RQ, fH62VV	6.7	15
C3102GR, fB32RR, fH62VI	6.2	16
C3102RR, fB32RQ, fH62VI	3.6	28
C3102GR, fB32RQ, fH62VV	3.4	30
C3102GG, fB32RQ, fH62VV	2.1	48
C3102GR, fB32RQ, fH62VI	1.8	56
C3102RR, fB32RR, fH62II	1.7	61
C3102GG, fB32RR, fH62VV	1.5	68
C3102GR, fB32RR, fH62II	0.83	121
C3102GG, fB32RR, fH62VI	0.78	129
C3102RR, fB32QQ, fH62VV	0.48	207
C3102RR, fB32RQ, fH62II	0.48	211
C3102RR, fB32QQ, fH62VI	0.257	389
C3102GR, fB32QQ, fH62VV	0.24	414
C3102GR, fB32RQ, fH62II	0.24	421
C3102GG, fB32RQ, fH62VI	0.22	448
C3102GR, fB32QQ, fH62VI	0.13	778
C3102GG, fB32RR, fH62II	0.10	969
C3102RR, fB32QQ, fH62II	0.034	2928
C3102GG, fB32QQ, fH62VV	0.030	3311
C3102GG, fB32RQ, fH62II	0.030	3368
C3102GR,fB32QQ, fH62II	0.017	5856
C3102GG, fB32QQ, fH62VI	0.016	6227
C3102GG, fB32QQ, fH62II	0.002	46851

## Box 1 Complement activation and regulation

Continuous, low level activation of C3 in plasma occurs through tickover; either spontaneous hydrolysis of the internal C3 thioester generates a C3b-like molecule, C3(H_2_O), or cleavage of C3 by plasma proteases generates C3b. These nascent, active molecules bind fB to form an AP convertase that cleaves C3 to C3b. In the AP amplification loop, C3b, either surface-bound or in the fluid phase, binds fB in a Mg^2+^-dependent manner and presents fB for cleavage by the plasma protease factor D (fD). The enzymatic fragment, Bb, remains attached to C3b and cleaves more C3 to C3b. Each C3 convertase can cleave many C3 molecules into C3b; thus, the AP amplification loop is a self-perpetuating pathway driving complement activation (Figure I). The C3bBb enzyme is naturally labile because Bb spontaneously dissociates from C3b with a half-life of about 90 s at 37 °C; however, binding of properdin (P) into the complex stabilises the enzyme and extends its half-life more than 10-fold.

Initiation of the CP and LP involves recognition of the target either through C1q binding target-bound antibody (CP) or MBL binding pathogen-specific sugar groups (LP). The associated protease units (C1s and mannan-binding lectin-associated serine protease 2 (MASP-2) respectively) then cleave C4 to C4b. Surface-bound C4b expresses a binding site for C2, leading to formation of the proenzyme C4b2. Cleavage of C2 by MASP-2 or C1s generates the active C3 convertase C4b2a. The AP amplifies the effects of activation of the CP and LP by generating AP C3 convertase from any C3b generated through these pathways. The activation pathway C3 convertases all cause C3b to shower down onto the target cell surface in the vicinity of the convertase, where it binds covalently to other C3b, the convertase, or other membrane structures in close proximity: C3b that binds the C3 convertase creates a new enzyme, the C5 convertase, C3bBbC3b or C4b2a3b, which cleaves C5 to C5b and C5a. C5a is a highly proinflammatory polypeptide which diffuses from the target surface to recruit other immune effectors, whereas C5b is the first structural building block of the lytic pore, the MAC. C5b sequentially binds the terminal pathway components, C6–C9, to assemble the macromolecular complex which forms holes through the membrane.

Self tissues are protected from accidental complement attack by proteins present in plasma and on cell membranes. These proteins are fH (AP-specific), C4b-binding protein (C4 bp; CP-specific), membrane cofactor protein (MCP, CD46), decay accelerating factor (DAF, CD55) and CR1 (CD35). These proteins restrict complement activation on self cells by either catalyzing proteolytic inactivation of C3b/C4b by fI (MCP, CR1, fH, C4 bp) or accelerating convertase dissociation (DAF, CR1, fH, C4 bp) [8].

## Box 2 Discovery and genotyping of AP complement polymorphisms

It has long been recognised that C3, fB and fH are polymorphic. The C3 R102G polymorphism was initially described at the protein level in 1967 as the C3S and C3F alleles; separated using prolonged agarose gel electrophoresis of fresh serum [77]. Similarly, the commonest factor B alleles BfS and BfF (now the R32W/Q polymorphism) were identified in 1972 using immunofixation after agarose gel electrophoresis of fresh serum [78]. Over a decade later, the common fH alleles FH*1 and FH*2 (now the Y402H polymorphism) were identified by isoelectric focusing in human serum after neuraminidase treatment under completely denaturing conditions [79].

During the 1990s, the knowledge, resources, and technologies emanating from the Human Genome Project resulted in the identification of most genetic variations in the human genome with frequencies >5%, including those in the AP complement genes. This understanding of human genetic variability resulted in an explosion of genetic studies, including genome wide association studies and candidate gene approaches, that have revealed association of complement genetic variations with disease and uncovered the important genotype–phenotype correlations discussed in this review.

Current methods for genotyping genetic variations in complement components do not differ from genotyping of any other gene. Genomic DNA is extracted from saliva, oral cavity mucosa or blood cells and analysed using a wide range of platforms based on DNA sequencing, DNA hybridization or mass spectrometry technologies.

Information related to genetic variations in complement genes can be found in several human genome databases in the public domain, including, the dbSNP (http://www.ncbi.nlm.nih.gov/projects/SNP/), the 1000 Genomes (http://www.1000genomes.org/) and the Exome Variant Server (http://evs.gs.washington.edu/EVS/).

## References

[1] Ricklin D. (2010). Complement: a key system for immune surveillance and homeostasis. Nat. Immunol..

[2] Lachmann P.J. (2009). The amplification loop of the complement pathways. Adv. Immunol..

[3] Pangburn M.K. (1981). Formation of the initial C3 convertase of the alternative complement pathway. Acquisition of C3b-like activities by spontaneous hydrolysis of the putative thioester in native C3. J. Exp. Med..

[4] Fearon D.T. (1978). Regulation by membrane sialic acid of beta1H-dependent decay-dissociation of amplification C3 convertase of the alternative complement pathway. Proc. Natl. Acad. Sci. U.S.A..

[5] Jozsi M., Zipfel P.F. (2008). Factor H family proteins and human diseases. Trends Immunol..

[6] Fritsche L.G. (2010). An imbalance of human complement regulatory proteins *CFHR1*, *CFHR3* and factor H influences risk for age-related macular degeneration (AMD). Hum. Mol. Genet..

[7] Morgan B.P., Meri S. (1994). Membrane proteins that protect against complement lysis. Springer Semin. Immunopathol..

[8] Morgan B.P., Harris C.L. (1999). Complement Regulatory Proteins.

[9] Lotery A. (2007). Burden of illness, visual impairment and health resource utilisation of patients with neovascular age-related macular degeneration: results from the UK cohort of a five-country cross-sectional study. Br. J. Ophthalmol..

[10] Haines J.L. (2005). Complement factor H variant increases the risk of age-related macular degeneration. Science.

[11] Edwards A.O. (2005). Complement factor H polymorphism and age-related macular degeneration. Science.

[12] Klein R.J. (2005). Complement factor H polymorphism in age-related macular degeneration. Science.

[13] Hageman G.S. (2005). A common haplotype in the complement regulatory gene factor H (*HF1/CFH*) predisposes individuals to age-related macular degeneration. Proc. Natl. Acad. Sci. U.S.A..

[14] Hughes A.E. (2006). A common *CFH* haplotype, with deletion of *CFHR1* and *CFHR3*, is associated with lower risk of age-related macular degeneration. Nat. Genet..

[15] Abarrategui-Garrido C. (2009). Characterization of complement factor H-related (*CFHR*) proteins in plasma reveals novel genetic variations of *CFHR1* associated with atypical hemolytic uremic syndrome. Blood.

[16] Martinez-Barricarte R. (2012). Relevance of complement factor H-related 1 (*CFHR1*) genotypes in age-related macular degeneration. Invest. Ophthalmol. Vis. Sci..

[17] Gold B. (2006). Variation in factor B (BF) and complement component 2 (C2) genes is associated with age-related macular degeneration. Nat. Genet..

[18] Yates J.R. (2007). Complement C3 variant and the risk of age-related macular degeneration. N. Engl. J. Med..

[19] Fagerness J.A. (2009). Variation near complement factor I is associated with risk of advanced AMD. Eur. J. Hum. Genet..

[20] Pickering M.C., Cook H.T. (2008). Translational mini-review series on complement factor H: renal diseases associated with complement factor H: novel insights from humans and animals. Clin. Exp. Immunol..

[21] Pickering M.C. (2007). Spontaneous hemolytic uremic syndrome triggered by complement factor H lacking surface recognition domains. J. Exp. Med..

[22] Abrera-Abeleda M.A. (2011). Allelic variants of complement genes associated with dense deposit disease. J. Am. Soc. Nephrol..

[23] Caprioli J. (2003). Complement factor H mutations and gene polymorphisms in haemolytic uraemic syndrome: the C-257T, the A2089G and the G2881T polymorphisms are strongly associated with the disease. Hum. Mol. Genet..

[24] Esparza-Gordillo J. (2005). Predisposition to atypical hemolytic uremic syndrome involves the concurrence of different susceptibility alleles in the regulators of complement activation gene cluster in 1q32. Hum. Mol. Genet..

[25] Manuelian T. (2003). Mutations in factor H reduce binding affinity to C3b and heparin and surface attachment to endothelial cells in hemolytic uremic syndrome. J. Clin. Invest..

[26] Rodriguez de Cordoba S., Goicoechea de Jorge E. (2008). Translational mini-review series on complement factor H: genetics and disease associations of human complement factor H. Clin. Exp. Immunol..

[27] Goicoechea de Jorge E. (2007). Gain-of-function mutations in complement factor B are associated with atypical hemolytic uremic syndrome. Proc. Natl. Acad. Sci. U.S.A..

[28] Fremeaux-Bacchi V. (2008). Mutations in complement C3 predispose to development of atypical hemolytic uremic syndrome. Blood.

[29] Rodriguez de Cordoba S. (2011). Lessons from functional and structural analyses of disease-associated genetic variants in the complement alternative pathway. Biochim. Biophys. Acta.

[30] Martinez-Barricarte R. (2008). The complement factor H R1210C mutation is associated with atypical hemolytic uremic syndrome. J. Am. Soc. Nephrol..

[31] Scholl H.P. (2008). Systemic complement activation in age-related macular degeneration. PLoS ONE.

[32] Reynolds R. (2009). Plasma complement components and activation fragments: associations with age-related macular degeneration genotypes and phenotypes. Invest. Ophthalmol. Vis. Sci..

[33] Hecker L.A. (2010). Genetic control of the alternative pathway of complement in humans and age-related macular degeneration. Hum. Mol. Genet..

[34] Montes T. (2009). Functional basis of protection against age-related macular degeneration conferred by a common polymorphism in complement factor B. Proc. Natl. Acad. Sci. U.S.A..

[35] Tortajada A. (2009). The disease-protective complement factor H allotypic variant Ile62 shows increased binding affinity for C3b and enhanced cofactor activity. Hum. Mol. Genet..

[36] Pechtl I.C. (2011). Disease-associated N-terminal complement factor H mutations perturb cofactor and decay-accelerating activities. J. Biol. Chem..

[37] Heurich M. (2011). Common polymorphisms in C3, factor B, and factor H collaborate to determine systemic complement activity and disease risk. Proc. Natl. Acad. Sci. U.S.A..

[38] Sjoberg A.P. (2007). The factor H variant associated with age-related macular degeneration (His-384) and the non-disease-associated form bind differentially to C-reactive protein, fibromodulin, DNA, and necrotic cells. J. Biol. Chem..

[39] Mihlan M. (2009). Monomeric CRP contributes to complement control in fluid phase and on cellular surfaces and increases phagocytosis by recruiting factor H. Cell Death Differ..

[40] Clark S.J. (2006). His-384 allotypic variant of factor H associated with age-related macular degeneration has different heparin binding properties from the non-disease-associated form. J. Biol. Chem..

[41] Herbert A.P. (2007). Structure shows that a glycosaminoglycan and protein recognition site in factor H is perturbed by age-related macular degeneration-linked single nucleotide polymorphism. J. Biol. Chem..

[42] Prosser B.E. (2007). Structural basis for complement factor H linked age-related macular degeneration. J. Exp. Med..

[43] Clark S.J. (2010). Impaired binding of the age-related macular degeneration-associated complement factor H 402H allotype to Bruch's membrane in human retina. J. Biol. Chem..

[44] Weismann D. (2011). Complement factor H binds malondialdehyde epitopes and protects from oxidative stress. Nature.

[45] Alper C.A. (1986). Complement genes of the major histocompatibility complex (complotypes), extended haplotypes and disease markers. Biochem. Soc. Symp..

[46] Kardys I. (2006). A common polymorphism in the complement factor H gene is associated with increased risk of myocardial infarction: the Rotterdam Study. J. Am. Coll. Cardiol..

[47] Messias-Reason I.J. (2003). Complement C3 F and BF S allotypes are risk factors for Chagas disease cardiomyopathy. Tissue Antigens.

[48] Papiha S.S. (1991). Factor B (BF) allotypes and multiple sclerosis in north-east England. Hum. Hered..

[49] Hagglof B. (1986). Studies of HLA, factor B (Bf), complement C2 and C4 haplotypes in type 1 diabetic and control families from northern Sweden. Hum. Hered..

[50] Finn J.E., Mathieson P.W. (1993). Molecular analysis of C3 allotypes in patients with nephritic factor. Clin. Exp. Immunol..

[51] Andrews P.A. (1995). Molecular analysis of C3 allotypes related to transplant outcome in human renal allografts. Transplantation.

[52] Rambausek M. (1987). Genetic polymorphism of C3 and Bf in IgA nephropathy. Nephrol. Dial. Transplant..

[53] Finn J.E. (1994). Molecular analysis of C3 allotypes in patients with systemic vasculitis. Nephrol. Dial. Transplant..

[54] Harold D. (2009). Genome-wide association study identifies variants at CLU and PICALM associated with Alzheimer's disease. Nat. Genet..

[55] Lambert J.C. (2009). Genome-wide association study identifies variants at CLU and CR1 associated with Alzheimer's disease. Nat. Genet..

[56] Fearon D.T., Carter R.H. (1995). The CD19/CR2/TAPA-1 complex of B lymphocytes: linking natural to acquired immunity. Annu. Rev. Immunol..

[57] Ahearn J.M., Fearon D.T. (1989). Structure and function of the complement receptors CR1 (CD35) and CR2 (CD21). Adv. Immunol..

[58] Keenan B.T. (2012). A coding variant in CR1 interacts with APOE-{varepsilon}4 to influence cognitive decline. Hum. Mol. Genet..

[59] Thambisetty M. (2010). Association of plasma clusterin concentration with severity, pathology, and progression in Alzheimer disease. Arch. Gen. Psychiatry.

[60] Szymanski M. (2011). Alzheimer's risk variants in the Clusterin gene are associated with alternative splicing. Transl. Psychiatry.

[61] Xing Y.Y. (2012). Blood clusterin levels, rs9331888 polymorphism, and the risk of Alzheimer's disease. J. Alzheimers Dis..

[62] Kurreeman F.A. (2007). A candidate gene approach identifies the TRAF1/C5 region as a risk factor for rheumatoid arthritis. PLoS Med..

[63] Zhernakova A. (2011). Meta-analysis of genome-wide association studies in celiac disease and rheumatoid arthritis identifies fourteen non-HLA shared loci. PLoS Genet..

[64] Morgan B.P. (1988). Measurement of terminal complement complexes in rheumatoid arthritis. Clin. Exp. Immunol..

[65] Wang Q. (2011). Identification of a central role for complement in osteoarthritis. Nat. Med..

[66] Jeong J.C. (2011). Association of complement 5 genetic polymorphism with renal allograft outcomes in Korea. Nephrol. Dial. Transplant..

[67] Song J.J. (2011). Plasma carboxypeptidase B downregulates inflammatory responses in autoimmune arthritis. J. Clin. Invest..

[68] Nishiguchi K.M. (2012). C9-R95X polymorphism in patients with neovascular age-related macular degeneration. Invest. Ophthalmol. Vis. Sci..

[69] Ermini L. (2011). Complement polymorphisms: geographical distribution and relevance to disease. Immunobiology.

[70] de Vries R.R. (1979). Genetic control of survival in epidemics. J. Immunogenet..

[71] Davila S. (2010). Genome-wide association study identifies variants in the *CFH* region associated with host susceptibility to meningococcal disease. Nat. Genet..

[72] Harrison L.H. (2009). Global epidemiology of meningococcal disease. Vaccine.

[73] Haralambous E. (2006). Factor H, a regulator of complement activity, is a major determinant of meningococcal disease susceptibility in UK Caucasian patients. Scand. J. Infect. Dis..

[74] Madico G. (2006). The meningococcal vaccine candidate GNA1870 binds the complement regulatory protein factor H and enhances serum resistance. J. Immunol..

[75] Woehrl B. (2011). Complement component 5 contributes to poor disease outcome in humans and mice with pneumococcal meningitis. J. Clin. Invest..

[76] Hakobyan S. (2010). Variant-specific quantification of factor H in plasma identifies null alleles associated with atypical hemolytic uremic syndrome. Kidney Int..

[77] Wieme R.J., Demeulenaere L. (1967). Genetically determined electrophoretic variant of the human complement component C′3. Nature.

[78] Alper C.A. (1972). Genetic polymorphism in human glycine-rich beta-glycoprotein. J. Exp. Med..

[79] Rodriguez de Cordoba S., Rubinstein P. (1984). Genetic polymorphism of human factor H (beta 1H). J. Immunol..

